# Intermittent low platelet counts hampering diagnosis of X-linked thrombocytopenia in children: report of two unrelated cases and a novel mutation in the gene coding for the Wiskott-Aldrich syndrome protein

**DOI:** 10.1186/s12887-017-0897-6

**Published:** 2017-06-22

**Authors:** Samuel Souza Medina, Lúcia Helena Siqueira, Marina Pereira Colella, Gabriela Goes Yamaguti-Hayakawa, Bruno Kosa Lino Duarte, Maria Marluce Dos Santos Vilela, Margareth Castro Ozelo

**Affiliations:** 1 Instituto Nacional de Ciência e Tecnologia do Sangue, Hemocentro de Campinas, University of Campinas (Unicamp), Rua Carlos Chagas 480, Cidade Universitária “Zeferino Vaz”, Campinas, SP 13.083-878 Brazil; 2 Centro de Investigação em Pediatria-CIPED, Faculty of Medical Sciences, University of Campinas (Unicamp), Rua Tessália Vieira de Camargo, 126, Cidade Universitária “Zeferino Vaz”, Campinas, SP 13.083-887 Brazil; 3 Department of Internal Medicine, Faculty of Medical Sciences, University of Campinas (Unicamp), Rua Tessália Vieira de Camargo, 126, Cidade Universitária “Zeferino Vaz”, Campinas, SP 13.083-887 Brazil

**Keywords:** Immune thrombocytopenia (ITP), thrombocytopenia, Wiskott-Aldrich syndrome (WAS), Wiskott-Aldrich syndrome protein (WASP), X-linked thrombocytopenia (XLT)

## Abstract

**Background:**

Thrombocytopenia can occur in different circumstances during childhood and although immune thrombocytopenia is its most frequent cause, it is important to consider other conditions, especially when there is a persistent or recurrent low platelet count. We report two cases of intermittent thrombocytopenia, previously misdiagnosed as immune thrombocytopenia.

**Cases presentation:**

Both cases described were boys who presented with an intermittent pattern of thrombocytopenia, with a persistently low mean platelet volume. In both patients, peripheral blood smear revealed small platelets and flow cytometry showed low expression of Wiskott-Aldrich syndrome protein (WASP) in leucocytes. Molecular analysis of the first case identified a mutation in exon 2 of the gene coding for WASP, leading to a p.Thr45Met amino acid change and confirming the diagnosis of X-linked thrombocytopenia. In the second case, a novel missense mutation in exon 2 of the gene coding for WASP was detected, which resulted in a p.Pro58Leu amino acid change.

**Conclusion:**

These two rare presentations of thrombocytopenia highlight the importance of evaluating the peripheral blood smear in the presence of recurrent or persistent thrombocytopenia and show that failing to do so can lead to misdiagnoses. Since thrombocytopenia may be found in pediatric outpatient clinic, increased awareness among general pediatricians will help to improve the differential diagnosis of this condition.

## Background

Children may occasionally present with thrombocytopenia, which is usually suspected in the presence of bleeding symptoms or even observed during routine evaluation in the asymptomatic patient. Immune-mediated destruction of platelets is the major mechanism involved in this event, and its most common cause is immune thrombocytopenia (ITP) [1]. The majority of ITP cases are self-limited, but about 25% of those are expected to become chronic [2]. Thus, when evaluating a case of persistent thrombocytopenia, the pediatrician should consider other diagnoses, including inherited platelet disorders.

Inherited thrombocytopenia comprises several distinct conditions, which can be classified according to platelet size [3]. Although rare, the presence of small platelets is consistently related to impaired expression of Wiskott-Aldrich syndrome protein (WASP), seen in both X-linked thrombocytopenia (XLT) and Wiskott-Aldrich syndrome (WAS) [4, 5]. In this manuscript, we describe two unrelated patients with an intermittent pattern of thrombocytopenia, who were previously diagnosed as ITP and further confirmed as XLT. We also present the diagnostic approach we carried out in both cases, which included gene sequencing and protein expression analysis, but we emphasize the importance of the assessment of the peripheral blood smear, which can be easily performed by any clinician.

## Cases presentation

### Methods

The confirmation of XLT diagnosis was based on the presence of microthrombocytopenia with low mean platelet volume (MPV) and on the reduced expression of WASP.

### Flow-cytometric analysis of WASP

Intracellular WASP expression was evaluated in 200 μl of peripheral whole blood, using Fix&Perm® Cell Permeabilization Kit (AN DER GRUB Bio Research GmbH) according to the manufacturer’s recommendations. Cells were incubated with 0.6 μg of mouse monoclonal anti-human WASP-FITC antibody (B-9) (Santa Cruz Biotechnology, Inc.), or isotype-matched control mouse IgG2a-FITC antibody Santa Cruz Biotechnology, Inc.), at room temperature for 15 min. Stained cells were analyzed with a FACSCanto™ flow cytometer and the CellQuest software (Becton Dickinson Immunocytometry Systems).

### Mutation analysis

WASP gene was amplified from genomic DNA isolated from leucocytes as described [6], and underwent direct sequencing.

### Patient 1

**Fig. 1:**
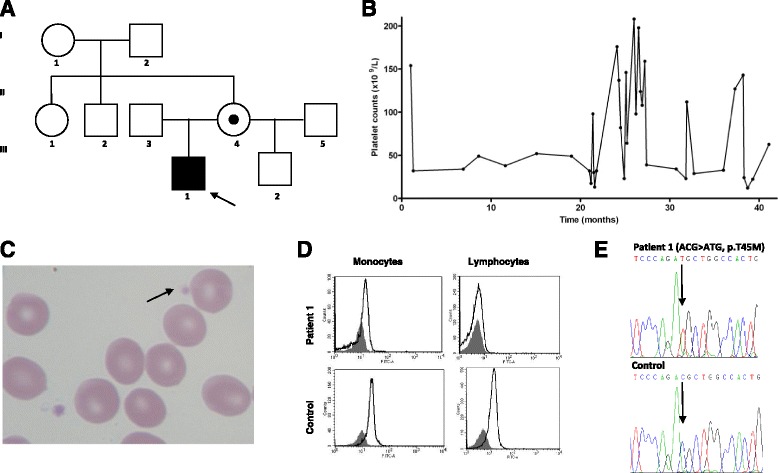
Patient 1. **a** Pedigree. The proband (III-1) is the only affected family member with X-linked thrombocytopenia. Mother is indicated as carrier. **b** Patient’s platelet count profile with intermittent thrombocytopenia without any clinically detectable triggering factor. **c** Patient’s peripheral blood smear with two platelets, one normal sized and a small platelet (*arrow*). **d** Wiskott-Aldrich syndome protein (WASP) expression analyzed by flow cytometry in monocytes and lymphocytes. In comparison to normal control, patient 1 showed reduced expression of WASP in both monocytes and lymphocytes (*white histograms*). *Gray histograms* represent isotype negative control antibody. **e** WASP gene sequencing from patient 1, with a C > T missense mutation in exon 2

Patient 1 was an 18-month-old boy with a Caucasian ethnic background who was referred to our service with a history of spontaneous mucocutaneous bleeds since he was nine months old. During his initial evaluation, before coming to us, complete blood count showed low platelet count, and the patient was diagnosed as having ITP. There was no abnormality either on child development or on physical examination. Family history of bleeding was uneventful. At that time, he was the only child of nonconsanguineous parents (Fig. 1a). During follow-up, he presented a spontaneous non-sustained increase in platelet counts. During one of the periods of thrombocytopenia, the patient was prescribed a short course of prednisone, but showed no response. When he first came to evaluation in our center, the initial laboratory investigation showed low platelet count (32 × 10^9^/L) and mild microcytic anemia, without any other abnormalities. Notably, we also observed persistently low MPV, ranging from 5.6 to 6.5 fL (normal range: 7.2–11.1 fL), and his peripheral blood smear showed two distinct platelet populations, one normal and another with markedly reduced size (Fig. 1c). In the following months, we checked the platelet count at weekly intervals and confirmed the existence of thrombocytopenia with an intermittent pattern, with platelet counts ranging from 12 to 208 × 10^9^/L (Fig. 1b). The combination of low MPV and intermittent pattern of thrombocytopenia suggested the diagnosis of XLT. To confirm it, we assessed WASP expression in peripheral blood cells by flow cytometry, and screened the WASP gene for mutations. We identified a lower expression of the protein in the patient’s peripheral blood leukocytes, when compared to a healthy control (Fig. 1d). A missense mutation on exon 2 of the WASP gene, resulting in a p.Thr45Met amino acid change, was detected (Fig. 1e). He never presented eczema, neutropenia or recurrent infections. Regarding laboratory data, the patient had normal leucocytes count, with normal subpopulations of lymphocytes, and normal immunoglobulin levels. All this together confirmed the diagnosis of XLT. His mother was heterozygous for the same mutation; the mutation was not found in the maternal grandparents, confirming the occurrence of a new mutation in this family. The mother had a second boy with normal platelet counts, whose genotype confirmed absence of the mutation in the WASP gene. We have been managing this patient’s thrombocytopenia episodes with prophylactic administration of antifibrinolytic agents. No major bleeding has occurred and no platelet transfusions have been necessary so far.

### Patient 2

**Fig. 2:**
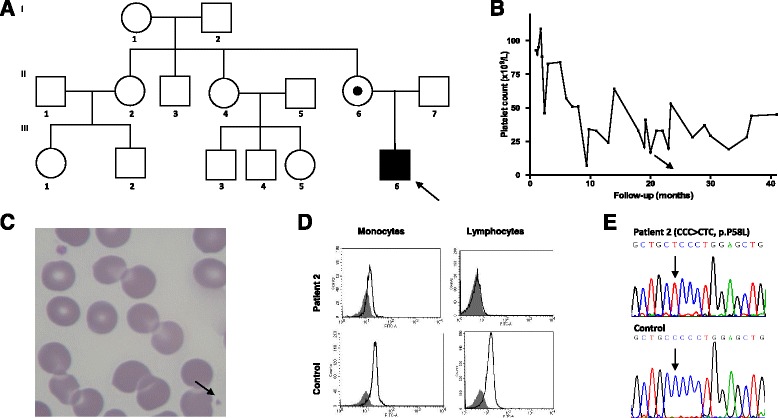
Patient 2. **a** Pedigree. X-linked thrombocytopenia was diagnosed only in the proband (III-6), and his mother was heterozygous for the mutation in Wiskott-Aldrich syndome protein (WASP) gene. **b** Patient’s platelet counts with intermittent thrombocytopenia pattern not associated to infection or any other triggering factor. **c** Patient’s peripheral blood smear showing a normal sized and a small platelet (*arrow*). **d** Reduced expression of WASP in monocytes and lymphocytes from patient 2 compared to normal control (*white histograms*) by flow cytometry. Isotype negative control antibody is shown in gray. **e** Sequencing of the WASP gene from patient 2, showing a C > T nucleotide change in exon 2

Patient 2 was an afro-descendent boy who was referred to us when he was 2 years old, with suspected diagnosis of ITP. During the neonatal period, at 15 days of age, he was diagnosed with pertussis, and a complete blood count revealed thrombocytopenia for the first time. Since then, his platelet counts ranged from 7 to 109 × 10^9^/L (Fig. 2b), with an unremarkable bleeding history, except for bruises after trauma. No family history of bleeding or consanguinity was reported (Fig. 2a). In the first 6 months of follow-up at our service, he presented with platelet counts ranging from 37 to 53 × 10^9^/L, and with the presence of small platelets in the peripheral blood smear (Fig. 2c). MPV was persistently low, ranging from 6.0 to 6.2 fL. Due to these clinical and laboratory findings, we considered the diagnosis of XLT. WASP expression in peripheral leukocytes of this patient was lower than in a healthy control (Fig. 2d). Molecular analysis of the WASP gene revealed a missense mutation in exon 2, leading to p.Pro58Leu amino acid change (Fig. 2e). He never presented eczema, neutropenia or recurrent infections, excluding diagnosis of WAS. His mother was heterozygous for the mutation, which was absent in the maternal grandparents. During follow-up, due to his mild bleeding symptoms, no hemostatic treatment has been needed so far.

## Discussion

There is a broad differential diagnosis for thrombocytopenia in pediatric patients. However, ITP is often considered the main cause of this finding [1], which can occur in up to 1 per 20,000 children per year in the United States [7]. In this report, we described two cases of inherited thrombocytopenia previously misdiagnosed as ITP. Both cases presented small platelets with persistently low MPV, illustrating the importance of platelet morphology assessment by combining peripheral blood smear evaluation, and platelet parameters in the automated blood count.

In pediatric population, ITP is usually triggered by previous infection or vaccination, and by six months after the onset, 75% of cases are expected to achieve complete remission [2]. In this way, those cases presenting with persistent or recurrent thrombocytopenia should be carefully evaluated for alternative diagnoses.

A differential diagnosis of thrombocytopenia should always consider platelet size, as proposed in Table 1. In addition, further characteristics of clinical presentation, and laboratory findings, can contribute for diagnosis in cases with thrombocytopenia. Both the presence of giant platelets and Döhle-like inclusion bodies in neutrophils are consistent to an autosomal dominant disorder, known as MYH9-related macrothrombocytopenia. In contrast, presence of parental consanguinity increases the probability of autosomal recessive disorders, such as Bernard-Soulier syndrome. Furthermore, wide variation of platelet counts, severe thrombocytopenia in very young children (e.g. <20 × 10^9^/L), and the history of preceding infection are more frequently seen in patients with ITP.

**Table 1:**
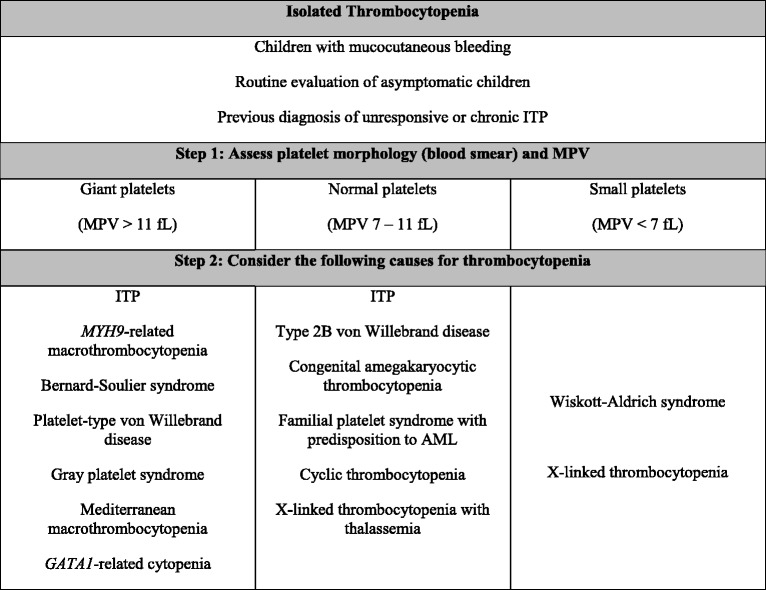
Suggested diagnostic approach for pediatric patients with isolated thrombocytopenia

*ITP* immune thrombocytopenia, *MPV* mean platelet volume, *AML* acute myeloid leukemia

WAS is a rare recessive X-linked disorder caused by mutations in the WASP gene, which encodes a protein involved in cellular signaling transduction to the actin cytoskeleton [5]. Recently, a primary immunodeficiency with the same features of WAS, inherited in an autosomal recessive manner, has been described in a female patient. It is caused by a mutation in the WIPF1 gene, which encodes the Wiskott-Aldrich interacting protein [8]. Mutations in the WASP gene are associated with a spectrum of clinical phenotypes including classic WAS, XLT, and X-linked neutropenia (XLN) [9, 10]. Classic WAS is characterized not only by microthrombocytopenia, but also by other clinical complications such as eczema, immunodeficiency, and increased risk of autoimmune disorders and malignancy [4]. Bleeding manifestations, such as petechiae and bruising, are usually present at birth, and recurrent infections are a common feature [11]. Thrombocytopenia is generally severe and is usually characterized by low platelet volume. In spite of the fact that there have been reports of normal platelet size in WAS patients [12], microthrombocytopenia is considered the hallmark finding of both classic WAS and XLT [11]. In addition, it is noteworthy that platelet count in WAS and XLT can be largely variable in some individual patients as previously shown by the authors of one multicenter review [13].

There is a very consistent phenotype-genotype correlation in WAS, and missense mutations in exons 1–3 of the WASP gene are most often identified in patients with milder forms, such as XLT [5]. A milder variant of XLT, characterized by intermittent thrombocytopenia, as seen in our cases, has been described in only three families so far [14, 15]. They presented missense mutations affecting amino acid residue 58 (Pro > Arg) in exon 2 [14], residue 481 (Ile > Asn) in exon 11 [14], and residue 56 (Ala > Thr) in exon 2 [15].

Interestingly, the mutation from patient 2 reported here affected WASP in the same residue as a previously described patient with intermittent thrombocytopenia (Pro58), but with the substitution by another amino acid (Pro > Leu). In addition, the p.Thr45Met mutation from patient 1 has not been previously associated with intermittent XLT, but with mild clinical forms of WAS [9]. Due to the absence of eczema and recurrent infections in patients with XLT, it is not surprising that these cases have been carried out as ITP. Thus, in children with persistent thrombocytopenia or even with intermittent pattern of low platelet count having constantly low MPV, the diagnosis of XLT may be considered in order to avoid an incorrect therapeutic approach.

## Conclusion

Differential diagnosis of persistent thrombocytopenia during childhood should always take into account the inherited thrombocytopenias. Among these disorders, evaluation of platelet morphology is an important parameter, which can be simply assessed through an easily reached examination such as peripheral blood smear. The presence of giant platelets, for instance, may suggest the diagnosis of MYH9-related macrothrombocytopenias, and Bernard-Soulier syndrome [3]. On the other hand, the presence of microthrombocytopenia is a pathognomonic sign of WASP-related disorders, including WAS and XLT. Particularly in XLT, some cases can present with an intermittent thrombocytopenia pattern, as showed in both cases reported here, and this can hamper the correct diagnosis. We believe that the frequency of XLT may be underestimated but this issue could be overcome with the use of a simple diagnostic approach.

## Abbreviations

ITP: Immune thrombocytopenia
MPV: Mean platelet volume
WAS: Wiskott-Aldrich syndrome
WASP: Wiskott-Aldrich syndrome protein
XLN: X-linked neutropenia
XLT: X-linked thrombocytopenia

## References

[1] Terrell DR, Beebe LA, Vesely SK, Neas BR, Segal JB, George JN (2010). The incidence of immune thrombocytopenic purpura in children and adults: a critical review of published reports. Am J Hematol.

[2] Rosthoj S, Hedlund-Treutiger I, Rajantie J, Zeller B, Jonsson OG, Elinder G (2003). Duration and morbidity of newly diagnosed idiopathic thrombocytopenic purpura in children: a prospective Nordic study of an unselected cohort. J Pediatr.

[3] Balduini CL, Pecci A, Noris P (2013). Diagnosis and management of inherited thrombocytopenias. Semin Thromb Hemost.

[4] Notarangelo LD, Miao CH, Ochs HD (2008). Wiskott-Aldrich syndrome. Curr Opin Hematol.

[5] Ochs HD (2009). Mutations of the Wiskott-Aldrich syndrome protein affect protein expression and dictate the clinical phenotypes. Immunol Res.

[6] Park SK, Kim CS, Song DK, Kim JY, Choi IJ, Kim DK (2007). A familial case of Wiskott-Aldrich syndrome with a hotspot mutation in exon 2 of the WAS Gene. J Korean Med Sci.

[7] Schultz CL, Mitra N, Schapira MM, Lambert MP (2014). Influence of the American Society of Hematology guidelines on the management of newly diagnosed childhood immune thrombocytopenia. JAMA Pediatr.

[8] Lanzi G, Moratto D, Vairo D, Masneri S, Delmonte O, Paganini T (2012). A novel primary human immunodeficiency due to deficiency in the WASP-interacting protein WIP. J Exp Med.

[9] Albert MH, Bittner TC, Nonoyama S, Notarangelo LD, Burns S, Imai K (2010). X-linked thrombocytopenia (XLT) due to WAS mutations: clinical characteristics, long-term outcome, and treatment options. Blood.

[10] Jin Y, Mazza C, Christie JR, Giliani S, Fiorini M, Mella P (2004). Mutations of the Wiskott-Aldrich Syndrome Protein (WASP): hotspots, effect on transcription, and translation and phenotype/genotype correlation. Blood.

[11] Ochs HD, Filipovich AH, Veys P, Cowan MJ, Kapoor N (2009). Wiskott-Aldrich syndrome: diagnosis, clinical and laboratory manifestations, and treatment. Biol Blood Marrow Transplant.

[12] Patel PD, Samanich JM, Mitchell WB, Manwani D (2011). A unique presentation of Wiskott-Aldrich syndrome in relation to platelet size. Pediatr Blood Cancer.

[13] Sullivan KE, Mullen CA, Blaese RM, Winkelstein JA (1994). A multiinstitutional survey of the Wiskott-Aldrich syndrome. J Pediatr.

[14] Notarangelo LD, Mazza C, Giliani S, D'Aria C, Gandellini F, Ravelli C (2002). Missense mutations of the WASP gene cause intermittent X-linked thrombocytopenia. Blood.

[15] Wada T, Itoh M, Maeba H, Toma T, Niida Y, Saikawa Y (2014). Intermittent X-linked thrombocytopenia with a novel WAS gene mutation. Pediatr Blood Cancer.

